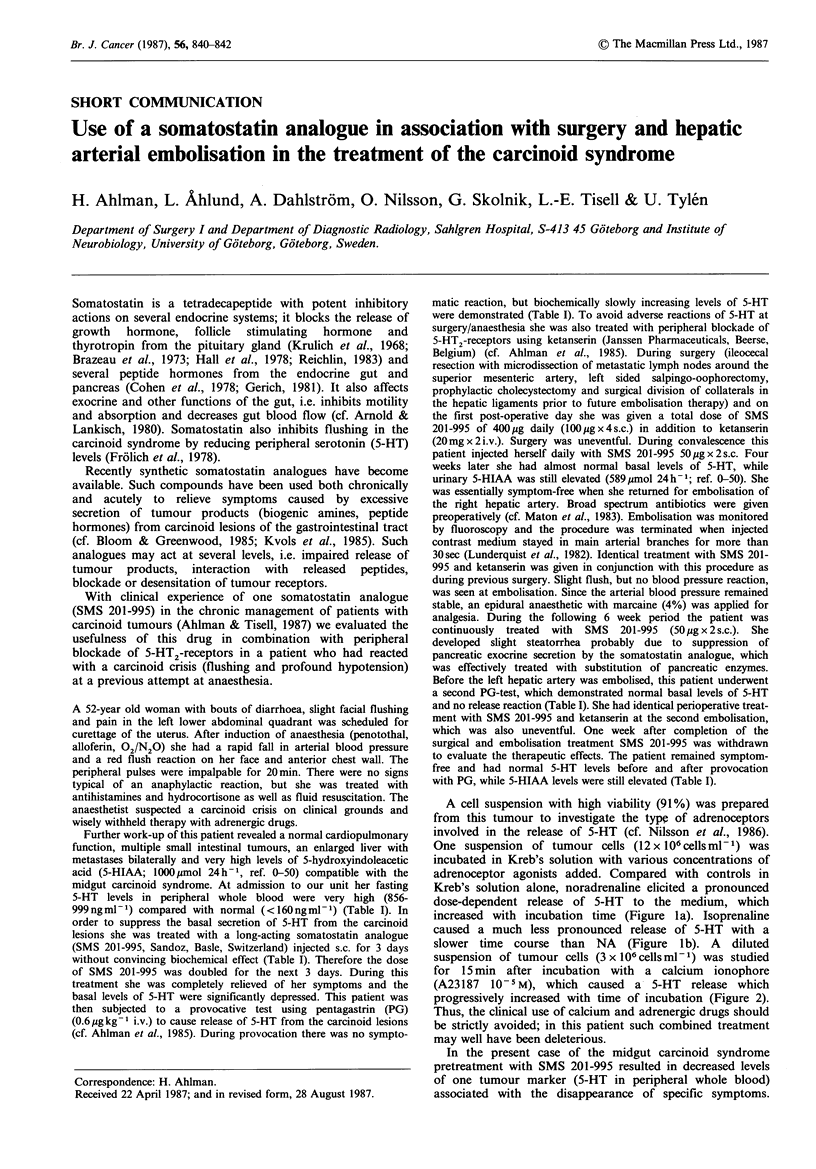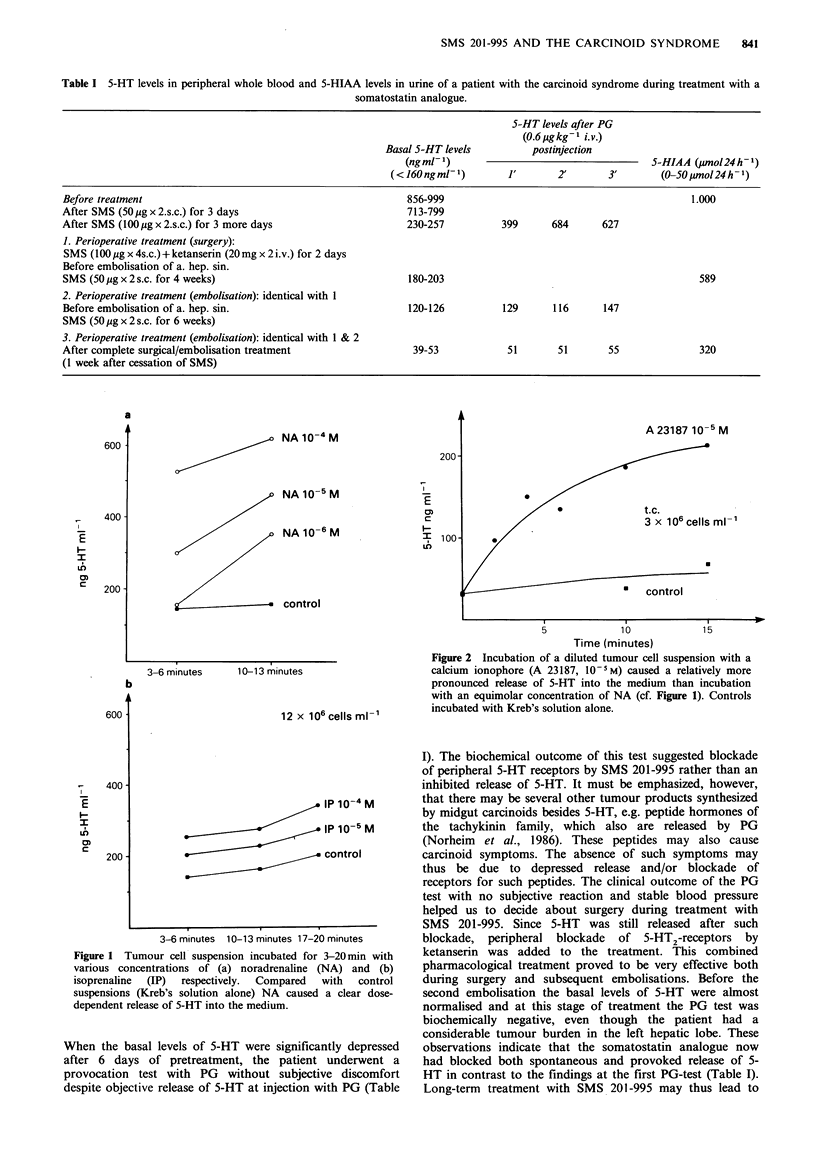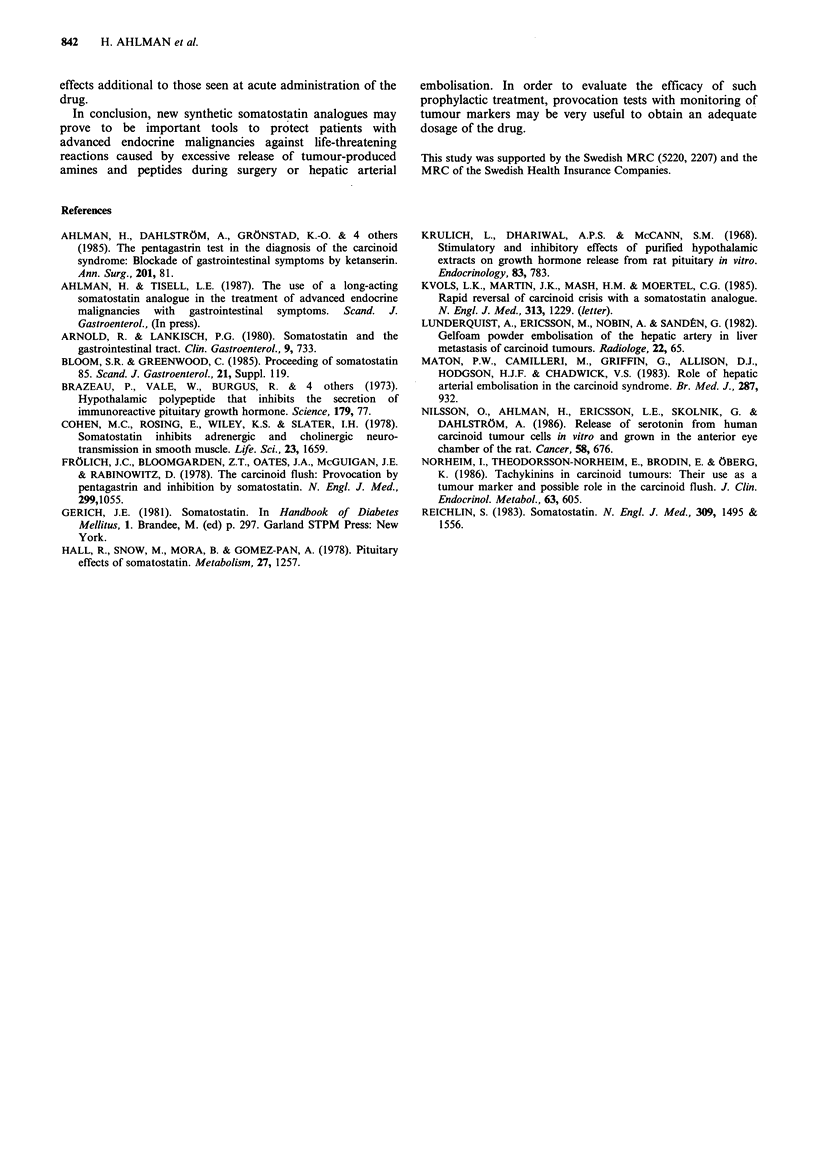# Use of a somatostatin analogue in association with surgery and hepatic arterial embolisation in the treatment of the carcinoid syndrome.

**DOI:** 10.1038/bjc.1987.302

**Published:** 1987-12

**Authors:** H. Ahlman, L. Ahlund, A. Dahlström, O. Nilsson, G. Skolnik, L. E. Tisell, U. Tylén

**Affiliations:** Department of Surgery I, Sahlgren Hospital, Göteborg, Sweden.


*Br. J. Cancer* (1987), **56**, 840–842 © The Macmillan Press Ltd., 1987

SHORT COMMUNICATION

### Use of a somatostatin analogue in association with surgery and hepatic arterial embolisation in the treatment of the carcinoid syndrome

H. Ahlman, L. Åhlund, A. Dahlström, O. Nilsson, G. Skolnik, L.-E. Tisell & U. Tylén

*Department of Surgery I and Department of Diagnostic Radiology, Sahlgren Hospital, S-413 45 Göteborg and Institute of Neurobiology, University of Göteborg, Göteborg, Sweden.*

Somatostatin is a tetradecapeptide with potent inhibitory actions on several endocrine systems; it blocks the release of growth hormone, follicle stimulating hormone and thyrotropin from the pituitary gland (Krulich *et al.*, 1968; Brazeau *et al.*, 1973; Hall *et al.*, 1978; Reichlin, 1983) and several peptide hormones from the endocrine gut and pancreas (Cohen *et al.*, 1978; Gerich, 1981). It also affects exocrine and other functions of the gut, i.e. inhibits motility and absorption and decreases gut blood flow (cf. Arnold & Lankisch, 1980). Somatostatin also inhibits flushing in the carcinoid syndrome by reducing peripheral serotonin (5-HT) levels (Frölich *et al.*, 1978).

Recently synthetic somatostatin analogues have become available. Such compounds have been used both chronically and acutely to relieve symptoms caused by excessive secretion of tumour products (biogenic amines, peptide hormones) from carcinoid lesions of the gastrointestinal tract (cf. Bloom & Greenwood, 1985; Kvols *et al.*, 1985). Such analogues may act at several levels, i.e. impaired release of tumour products, interaction with released peptides, blockade or desensitation of tumour receptors.

With clinical experience of one somatostatin analogue (SMS 201-995) in the chronic management of patients with carcinoid tumours (Ahlman & Tisell, 1987) we evaluated the usefulness of this drug in combination with peripheral blockade of 5-$HT_2$-receptors in a patient who had reacted with a carcinoid crisis (flushing and profound hypotension) at a previous attempt at anaesthesia.

A 52-year old woman with bouts of diarrhoea, slight facial flushing and pain in the left lower abdominal quadrant was scheduled for curettage of the uterus. After induction of anaesthesia (penotothal, alloferin, $O_2/N_2O$) she had a rapid fall in arterial blood pressure and a red flush reaction on her face and anterior chest wall. The peripheral pulses were impalpable for 20 min. There were no signs typical of an anaphylactic reaction, but she was treated with antihistamines and hydrocortisone as well as fluid resuscitation. The anaesthetist suspected a carcinoid crisis on clinical grounds and wisely withheld therapy with adrenergic drugs.

Further work-up of this patient revealed a normal cardiopulmonary function, multiple small intestinal tumours, an enlarged liver with metastases bilaterally and very high levels of 5-hydroxyindoleacetic acid (5-HIAA; 1000 $\mu$mol 24 $h^{-1}$, ref. 0–50) compatible with the midgut carcinoid syndrome. At admission to our unit her fasting 5-HT levels in peripheral whole blood were very high (856–999 ng $ml^{-1}$) compared with normal (<160 ng $ml^{-1}$) (Table I). In order to suppress the basal secretion of 5-HT from the carcinoid lesions she was treated with a long-acting somatostatin analogue (SMS 201-995, Sandoz, Basle, Switzerland) injected s.c. for 3 days without convincing biochemical effect (Table I). Therefore the dose of SMS 201-995 was doubled for the next 3 days. During this treatment she was completely relieved of her symptoms and the basal levels of 5-HT were significantly depressed. This patient was then subjected to a provocative test using pentagastrin (PG) (0.6 $\mu$g $kg^{-1}$ i.v.) to cause release of 5-HT from the carcinoid lesions (cf. Ahlman *et al.*, 1985). During provocation there was no symptomatic reaction, but biochemically slowly increasing levels of 5-HT were demonstrated (Table I). To avoid adverse reactions of 5-HT at surgery/anaesthesia she was also treated with peripheral blockade of 5-$HT_2$-receptors using ketanserin (Janssen Pharmaceuticals, Beerse, Belgium) (cf. Ahlman *et al.*, 1985). During surgery (ileocecal resection with microdissection of metastatic lymph nodes around the superior mesenteric artery, left sided salpingo-oophorectomy, prophylactic cholecystectomy and surgical division of collaterals in the hepatic ligaments prior to future embolisation therapy) and on the first post-operative day she was given a total dose of SMS 201-995 of 400 $\mu$g daily (100 $\mu$g × 4 s.c.) in addition to ketanserin (20 mg × 2 i.v.). Surgery was uneventful. During convalescence this patient injected herself daily with SMS 201-995 50 $\mu$g × 2 s.c. Four weeks later she had almost normal basal levels of 5-HT, while urinary 5-HIAA was still elevated (589 $\mu$mol 24 $h^{-1}$; ref. 0–50). She was essentially symptom-free when she returned for embolisation of the right hepatic artery. Broad spectrum antibiotics were given preoperatively (cf. Maton *et al.*, 1983). Embolisation was monitored by fluoroscopy and the procedure was terminated when injected contrast medium stayed in main arterial branches for more than 30 sec (Lunderquist *et al.*, 1982). Identical treatment with SMS 201-995 and ketanserin was given in conjunction with this procedure as during previous surgery. Slight flush, but no blood pressure reaction, was seen at embolisation. Since the arterial blood pressure remained stable, an epidural anaesthetic with marcaine (4%) was applied for analgesia. During the following 6 week period the patient was continuously treated with SMS 201-995 (50 $\mu$g × 2 s.c.). She developed slight steatorrhea probably due to suppression of pancreatic exocrine secretion by the somatostatin analogue, which was effectively treated with substitution of pancreatic enzymes. Before the left hepatic artery was embolised, this patient underwent a second PG-test, which demonstrated normal basal levels of 5-HT and no release reaction (Table I). She had identical perioperative treatment with SMS 201-995 and ketanserin at the second embolisation, which was also uneventful. One week after completion of the surgical and embolisation treatment SMS 201-995 was withdrawn to evaluate the therapeutic effects. The patient remained symptom-free and had normal 5-HT levels before and after provocation with PG, while 5-HIAA levels were still elevated (Table I).

A cell suspension with high viability (91%) was prepared from this tumour to investigate the type of adrenoceptors involved in the release of 5-HT (cf. Nilsson *et al.*, 1986). One suspension of tumour cells ($12 \times 10^6$ cells $ml^{-1}$) was incubated in Kreb's solution with various concentrations of adrenoceptor agonists added. Compared with controls in Kreb's solution alone, noradrenaline elicited a pronounced dose-dependent release of 5-HT to the medium, which increased with incubation time (Figure 1a). Isoprenaline caused a much less pronounced release of 5-HT with a slower time course than NA (Figure 1b). A diluted suspension of tumour cells ($3 \times 10^6$ cells $ml^{-1}$) was studied for 15 min after incubation with a calcium ionophore (A23187 $10^{-5}$ M), which caused a 5-HT release which progressively increased with time of incubation (Figure 2). Thus, the clinical use of calcium and adrenergic drugs should be strictly avoided; in this patient such combined treatment may well have been deleterious.

In the present case of the midgut carcinoid syndrome pretreatment with SMS 201-995 resulted in decreased levels of one tumour marker (5-HT in peripheral whole blood) associated with the disappearance of specific symptoms.

Correspondence: H. Ahlman.
Received 22 April 1987; and in revised form, 28 August 1987.

**Table I** 5-HT levels in peripheral whole blood and 5-HIAA levels in urine of a patient with the carcinoid syndrome during treatment with a somatostatin analogue.

| | *Basal 5-HT levels* (*ng ml*$^{-1}$) (*< 160 ng ml*$^{-1}$) | *5-HT levels after PG* (*0.6 μg kg*$^{-1}$ *i.v.*) *postinjection* 1′ | 2′ | 3′ | *5-HIAA* (*μmol 24 h*$^{-1}$) (*0–50 μmol 24 h*$^{-1}$) |
|---|---|---|---|---|---|
| *Before treatment* | 856-999 | | | | 1.000 |
| After SMS (50 μg × 2.s.c.) for 3 days | 713-799 | | | | |
| After SMS (100 μg × 2.s.c.) for 3 more days | 230-257 | 399 | 684 | 627 | |
| *1. Perioperative treatment* (*surgery*): | | | | | |
| SMS (100 μg × 4s.c.) + ketanserin (20 mg × 2 i.v.) for 2 days | | | | | |
| Before embolisation of a. hep. sin. | | | | | |
| SMS (50 μg × 2 s.c. for 4 weeks) | 180-203 | | | | 589 |
| *2. Perioperative treatment* (*embolisation*): identical with 1 | | | | | |
| Before embolisation of a. hep. sin. | 120-126 | 129 | 116 | 147 | |
| SMS (50 μg × 2 s.c. for 6 weeks) | | | | | |
| *3. Perioperative treatment* (*embolisation*): identical with 1 & 2 | | | | | |
| After complete surgical/embolisation treatment | 39-53 | 51 | 51 | 55 | 320 |
| (1 week after cessation of SMS) | | | | | |

**Figure 1** Tumour cell suspension incubated for 3–20 min with various concentrations of (a) noradrenaline (NA) and (b) isoprenaline (IP) respectively. Compared with control suspensions (Kreb's solution alone) NA caused a clear dose-dependent release of 5-HT into the medium.

**Figure 2** Incubation of a diluted tumour cell suspension with a calcium ionophore (A 23187, $10^{-5}$ M) caused a relatively more pronounced release of 5-HT into the medium than incubation with an equimolar concentration of NA (cf. **Figure 1**). Controls incubated with Kreb's solution alone.

When the basal levels of 5-HT were significantly depressed after 6 days of pretreatment, the patient underwent a provocation test with PG without subjective discomfort despite objective release of 5-HT at injection with PG (Table I). The biochemical outcome of this test suggested blockade of peripheral 5-HT receptors by SMS 201-995 rather than an inhibited release of 5-HT. It must be emphasized, however, that there may be several other tumour products synthesized by midgut carcinoids besides 5-HT, e.g. peptide hormones of the tachykinin family, which also are released by PG (Norheim *et al.*, 1986). These peptides may also cause carcinoid symptoms. The absence of such symptoms may thus be due to depressed release and/or blockade of receptors for such peptides. The clinical outcome of the PG test with no subjective reaction and stable blood pressure helped us to decide about surgery during treatment with SMS 201-995. Since 5-HT was still released after such blockade, peripheral blockade of $5\text{-HT}_2$-receptors by ketanserin was added to the treatment. This combined pharmacological treatment proved to be very effective both during surgery and subsequent embolisations. Before the second embolisation the basal levels of 5-HT were almost normalised and at this stage of treatment the PG test was biochemically negative, even though the patient had a considerable tumour burden in the left hepatic lobe. These observations indicate that the somatostatin analogue now had blocked both spontaneous and provoked release of 5-HT in contrast to the findings at the first PG-test (Table I). Long-term treatment with SMS 201-995 may thus lead to effects additional to those seen at acute administration of the drug.

In conclusion, new synthetic somatostatin analogues may prove to be important tools to protect patients with advanced endocrine malignancies against life-threatening reactions caused by excessive release of tumour-produced amines and peptides during surgery or hepatic arterial embolisation. In order to evaluate the efficacy of such prophylactic treatment, provocation tests with monitoring of tumour markers may be very useful to obtain an adequate dosage of the drug.

This study was supported by the Swedish MRC (5220, 2207) and the MRC of the Swedish Health Insurance Companies.

### References

AHLMAN, H., DAHLSTRÖM, A., GRÖNSTAD, K.-O. & 4 others (1985). The pentagastrin test in the diagnosis of the carcinoid syndrome: Blockade of gastrointestinal symptoms by ketanserin. *Ann. Surg.*, **201**, 81.

AHLMAN, H. & TISELL, L.E. (1987). The use of a long-acting somatostatin analogue in the treatment of advanced endocrine malignancies with gastrointestinal symptoms. *Scand. J. Gastroenterol.*, (In press).

ARNOLD, R. & LANKISCH, P.G. (1980). Somatostatin and the gastrointestinal tract. *Clin. Gastroenterol.*, **9**, 733.

BLOOM, S.R. & GREENWOOD, C. (1985). Proceeding of somatostatin 85. *Scand. J. Gastroenterol.*, **21**, Suppl. 119.

BRAZEAU, P., VALE, W., BURGUS, R. & 4 others (1973). Hypothalamic polypeptide that inhibits the secretion of immunoreactive pituitary growth hormone. *Science*, **179**, 77.

COHEN, M.C., ROSING, E., WILEY, K.S. & SLATER, I.H. (1978). Somatostatin inhibits adrenergic and cholinergic neurotransmission in smooth muscle. *Life. Sci.*, **23**, 1659.

FRÖLICH, J.C., BLOOMGARDEN, Z.T., OATES, J.A., MCGUIGAN, J.E. & RABINOWITZ, D. (1978). The carcinoid flush: Provocation by pentagastrin and inhibition by somatostatin. *N. Engl. J. Med.*, **299**,1055.

GERICH, J.E. (1981). Somatostatin. In *Handbook of Diabetes Mellitus*, **1**. Brandee, M. (ed) p. 297. Garland STPM Press: New York.

HALL, R., SNOW, M., MORA, B. & GOMEZ-PAN, A. (1978). Pituitary effects of somatostatin. *Metabolism*, **27**, 1257.

KRULICH, L., DHARIWAL, A.P.S. & MCCANN, S.M. (1968). Stimulatory and inhibitory effects of purified hypothalamic extracts on growth hormone release from rat pituitary *in vitro*. *Endocrinology*, **83**, 783.

KVOLS, L.K., MARTIN, J.K., MASH, H.M. & MOERTEL, C.G. (1985). Rapid reversal of carcinoid crisis with a somatostatin analogue. *N. Engl. J. Med.*, **313**, 1229. (*letter*).

LUNDERQUIST, A., ERICSSON, M., NOBIN, A. & SANDÉN, G. (1982). Gelfoam powder embolisation of the hepatic artery in liver metastasis of carcinoid tumours. *Radiologe*, **22**, 65.

MATON, P.W., CAMILLERI, M., GRIFFIN, G., ALLISON, D.J., HODGSON, H.J.F. & CHADWICK, V.S. (1983). Role of hepatic arterial embolisation in the carcinoid syndrome. *Br. Med. J.*, **287**, 932.

NILSSON, O., AHLMAN, H., ERICSSON, L.E., SKOLNIK, G. & DAHLSTRÖM, A. (1986). Release of serotonin from human carcinoid tumour cells *in vitro* and grown in the anterior eye chamber of the rat. *Cancer*, **58**, 676.

NORHEIM, I., THEODORSSON-NORHEIM, E., BRODIN, E. & ÖBERG, K. (1986). Tachykinins in carcinoid tumours: Their use as a tumour marker and possible role in the carcinoid flush. *J. Clin. Endocrinol. Metabol.*, **63**, 605.

REICHLIN, S. (1983). Somatostatin. *N. Engl. J. Med.*, **309**, 1495 & 1556.